# Disruption of *SMIM1* causes the Vel− blood type

**DOI:** 10.1002/emmm.201302466

**Published:** 2013-04-15

**Authors:** Bryan A Ballif, Virginie Helias, Thierry Peyrard, Cécile Menanteau, Carole Saison, Nicole Lucien, Sébastien Bourgouin, Maude Le Gall, Jean-Pierre Cartron, Lionel Arnaud

**Affiliations:** 1 Department of Biology, University of VermontBurlington, VT, USA; 2 National Institute of Blood Transfusion (INTS)Paris, France; 3 National Reference Center for Blood Groups (CNRGS)Paris, France; 4 Centre de Recherche Biomédicale Bichat-Beaujon, UMR-S 773, Inserm, Université Paris Diderot, Sorbonne Paris CitéParis, France

**Keywords:** blood group, genotyping, mass spectrometry, membrane protein, Vel

## Abstract

Here, we report the biochemical and genetic basis of the Vel blood group antigen, which has been a vexing mystery for decades, especially as anti-Vel regularly causes severe haemolytic transfusion reactions. The protein carrying the Vel blood group antigen was biochemically purified from red blood cell membranes. Mass spectrometry-based *de novo* peptide sequencing identified this protein to be small integral membrane protein 1 (SMIM1), a previously uncharacterized single-pass membrane protein. Expression of *SMIM1* cDNA in Vel− cultured cells generated anti-Vel cell surface reactivity, confirming that *SMIM1* encoded the Vel blood group antigen. A cohort of 70 Vel− individuals was found to be uniformly homozygous for a 17 nucleotide deletion in the coding sequence of *SMIM1*. The genetic homogeneity of the Vel− blood type, likely having a common origin, facilitated the development of two highly specific DNA-based tests for rapid *Vel* genotyping, which can be easily integrated into blood group genotyping platforms. These results answer a 60-year-old riddle and provide tools of immediate assistance to all clinicians involved in the care of Vel− patients.

## INTRODUCTION

Blood types are defined by the presence or absence of specific antigens at the surface of red blood cells (RBCs) and are inherited characters that result from genetic polymorphisms at different loci (Daniels, 2009). Systematic efforts to identify all these polymorphisms have allowed important improvements in transfusion safety and obstetrics, especially with the recent development of high-throughput platforms for blood group genotyping (Reid, 2009). While the genetic underpinnings of most blood group antigens have been identified, a few have stubbornly eluded discovery in spite of intense efforts motivated by their clinical significance. Such has been the case for the Vel antigen whose corresponding alloantibody, anti-Vel, is regularly responsible for severe acute or delayed haemolytic transfusion reactions (HTRs) that may induce life-threatening kidney failure (for a review, see Daniels, 2002).

The existence of the Vel antigen was recognized in 1952 by Sussman and Miller who found an alloantibody with a novel specificity in the serum of Mrs. ‘Vel’ who suffered an acute HTR (Sussman & Miller, 1952). The Vel antigen is expressed on the RBCs of most people so that Sussman and Miller identified only four individuals whose RBCs did not react with the serum of Mrs. ‘Vel’ by screening 10,000 blood donors with the O blood type from New York city (Sussman & Miller, 1952). This frequency of 0.0004 for individuals lacking the Vel antigen was confirmed by several large studies conducted in different parts of the world (Daniels, 2002), even though regional differences exist. Thus the analysis of 91,605 blood samples from northern Sweden identified 52 Vel− samples (frequency 0.0006; Cedergren et al, 1976) while the analysis of 62,586 blood samples from southern Wales identified 14 Vel− samples (frequency 0.0002; Gale et al, 1988). Vel− blood is one of the most difficult blood types to supply in many countries. This is partly due to the rarity of the Vel− blood type but also to the lack of systematic screening for the Vel− blood type in blood donors. When autologous transfusion is not possible, the supply of Vel− blood must be managed with help from national or international rare blood banks (Nance, 2009). However, not all requests for Vel− blood in Europe and North America can currently be filled.

The Vel− blood type is thought to be inherited as a recessive trait and is typically unveiled when Vel− individuals develop anti-Vel after transfusion or pregnancy. While the Vel− blood type is rare, many examples of anti-Vel have been reported (for a detailed review, see Sussman, 1962) such that now the identification of additional anti-Vel is rarely the subject of new reports. However, the clinical surfacing of an anti-Vel is dreaded by immunohaematologists. Anti-Vel is well known for its aggressive haemolytic activity *in vivo* as well as for its notorious recalcitrance *in vitro*. Notably, anti-Vel masquerades as a ‘high-titer, low-avidity’ antibody that can easily be missed when performing an antibody screen without appropriate methodology (Neppert et al, 1998; Storry & Mallory, 1994; Sweeney, 2006). Furthermore, some individuals appear to express very low levels of the Vel antigen that can be challenging to detect, especially since anti-Vel does not work well in adsorption-elution studies. In this case there is a risk of transfusing a patient having developed an anti-Vel with RBCs that were typed as Vel− but actually express the Vel antigen (even weakly).

Due to the high clinical significance of anti-Vel, and the inherent limitations of serological tests for the Vel antigen, intense efforts have been made toward finding the genetic basis of the Vel− blood type. However, all approaches to unravel the molecular basis of the Vel blood group antigen have proven unsuccessful. Here we describe the biochemical purification of the protein carrying the Vel antigen and its identification as SMIM1 by mass spectrometry-based sequencing. Furthermore, we report that a deletion in *SMIM1* is predominantly responsible for the Vel− blood type.

## RESULTS

### Characterization of the Vel antigen by Western blot analysis

**Figure 1:**
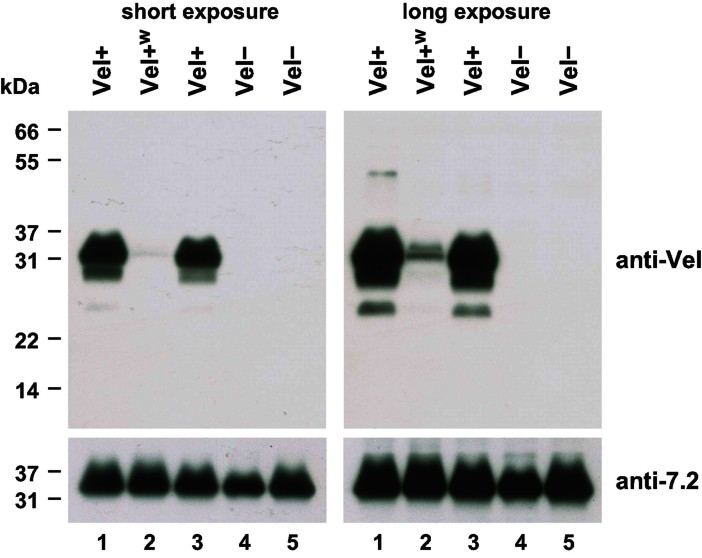
Anti-Vel Western blot of non-reduced RBC membrane extracts identifies a 32 kDa band present only in Vel+ individuals. RBC membrane extracts prepared from two Vel+ (lanes 1 and 3), one Vel+^w^ (lane 2), and two Vel− (lanes 4 and 5) subjects were resolved by non-reducing SDS–PAGE, and probed with an anti-Vel (top panels). A long exposure time was required to detect the 32 kDa reactive band in the Vel+^w^ subject (top right panel), who later appeared to be heterozygous for the 17 nt deletion in *SMIM1*. The same blot was reprobed with anti-7.2b (bottom panels).

Toward the identification of the molecular basis of the Vel antigen, we first performed an anti-Vel Western blot of RBC membrane extracts. For this purpose, we affinity purified a potent anti-Vel that was still present in the serum of a Vel− patient 27 years after receiving an incompatible transfusion. As shown in Fig 1, this purified anti-Vel strongly detected a band of approximately 32 kDa in RBC membrane extracts prepared from Vel+ subjects (lanes 1 and 3) but not from Vel− subjects (lanes 4 and 5). We hypothesized that this 32 kDa band corresponded to the carrier of the Vel antigen. We also analyzed RBC membrane extracts prepared from Vel-weak (Vel+^w^) subjects, who are often challenging to type and are sometimes mistyped as Vel−. The 32 kDa band was barely detectable in Vel+^w^ subjects (Fig 1, lane 2), corroborating the hypothesis that this 32 kDa band corresponded to the Vel antigen carrier.

**Figure 2:**
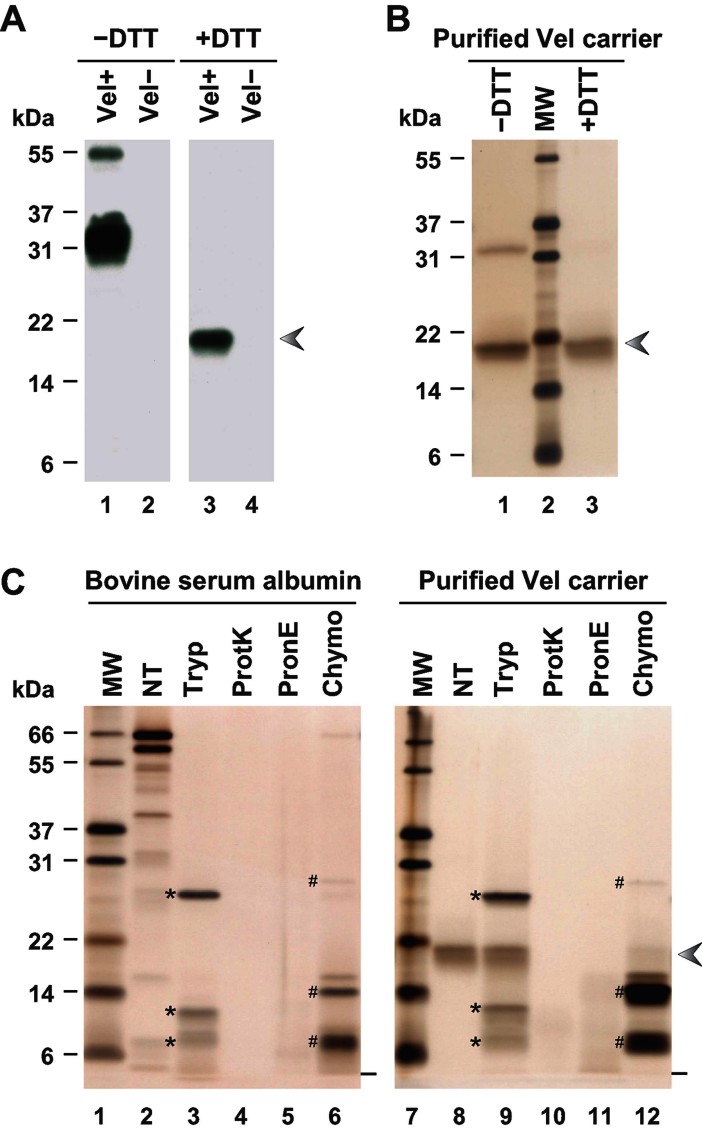
Purification and protease sensitivity of the 18 kDa reduced form of the Vel antigen carrier. A. RBC membrane extracts prepared from a Vel+ subject (lanes 1 and 3) and a Vel− subject (lanes 2 and 4) were resolved by SDS–PAGE under non-reducing (lanes 1 and 2) or reducing conditions (lanes 3 and 4), and probed with an anti-Vel. B. The 18 kDa material purified from a Vel+ RBC membrane extract was analyzed by SDS–PAGE, under non-reducing (lane 1) or reducing conditions (lane 3), and detected by silver staining. C. Bovine serum albumin (lanes 2–6) and the 18 kDa band purified from a Vel+ RBC membrane extract (lanes 8–12) were treated with trypsin (Tryp), proteinase K (ProtK), pronase E (PronE) or chymotrypsin (Chymo), or were left untreated (NT), and analyzed by reducing SDS–PAGE followed by silver staining. The electrophoretic migration fronts are indicated by dashes, trypsin and its autoproteolytic fragments by asterisks, and chymotrypsin and its autoproteolytic fragments by number signs. The arrowhead indicates the migration of the Vel antigen carrier under reducing conditions.

It is important to note that the aforementioned Western blots were performed without reducing agents such as dithiothreitol (DTT) as has been the tradition for RBC membrane protein analysis. Several months later, we serendipitously found that the Vel antigen carrier migrated at approximately 18 kDa when RBC membrane extracts were reduced with DTT prior to electrophoresis (Fig 2A, lane 3). These results suggested that the Vel antigen carrier was a relatively small membrane constituent capable of forming a molecular complex via disulphide bonds.

### Purification of the Vel antigen carrier by double electrophoresis

To purify the Vel antigen carrier, we decided to make use of its differential electrophoretic mobility under non-reducing and reducing conditions, *i.e.* 32 and 18 kDa, respectively. As a first purification step, RBC membrane extracts (prepared from random, Vel+ blood donors) were resolved by non-reducing polyacrylamide-gel electrophoresis (PAGE), and the material migrating at approximately 32 kDa was recovered by electro-elution. As a second purification step, this 32 kDa material was resolved by reducing PAGE, after which the material migrating at approximately 18 kDa was recovered. Fig 2B shows the effectiveness of this purification by double electrophoresis, given the apparent purity of an 18 kDa band upon reduction of the final product with DTT (lane 3). We recovered no 18 kDa band when we applied the same purification process to the RBC membrane extract prepared from a Vel− subject, indicating that this purified 18 kDa band was specific to Vel+ subjects. It also suggested that Vel− subjects may lack the corresponding protein. Interestingly, when the Vel-specific purified material was analyzed under non-reducing conditions, a portion of this material reemerged at 32 kDa (Fig 2B, lane 1), consistent with the 32 kDa band corresponding to a dimer of the 18 kDa monomeric form of the Vel antigen carrier.

We investigated the identity of the purified 18 kDa band by mass spectrometry after in-gel trypsin digestion. Only one candidate protein was confidently identified in two separate analyses and it corresponded to GAPR1 (also known as C9orf19 or GLIPR2). GAPR1 was originally characterized as a 17 kDa peripheral membrane protein that is associated with the Golgi apparatus (Eberle et al, 2002). GAPR1 was later shown to also be a secreted protein (Baxter et al, 2007). Although we demonstrated by Western blot analysis that GAPR1 was associated with the RBC membrane (the study of GAPR1 in RBCs will be described elsewhere), we found that GAPR1 was equally present in Vel+ and Vel− subjects, in contrast to the 18 kDa constituent that carries the Vel antigen. We hence concluded that GAPR1 was not the Vel antigen carrier but was a minute contaminant of the Vel-specific 18 kDa material that we had purified.

### Identification of the Vel antigen carrier protein by *de novo* sequencing

Given that GAPR1 was the only confidently identified protein from the mass spectrometry analysis of the in-gel tryptic digest of the Vel-specific 18 kDa band, we hypothesized that the protein carrying the Vel antigen might be resistant to proteolytic cleavage by trypsin. We tested this by digesting the purified material with several different proteases. Complete digestion was achieved with proteinase K (Fig 2C, lane 10) and pronase E (Fig 2C, lane 11), and at least partial digestion was achieved with chymotrypsin (Fig 2C, lane 12). These results confirmed the peptidic nature of the Vel-specific 18 kDa band. In contrast, this 18 kDa band appeared to be largely resistant to trypsin (Fig 2C, lane 9), which explained *a posteriori* the failure of our previous attempts to identify the Vel antigen carrier protein by mass spectrometry analysis after in-gel trypsin digest. Hence, we digested the purified 18 kDa band with chymotrypsin prior to mass spectrometry analysis. Disappointingly, analysis of the mass spectrometry data using the standard program SEQUEST (Eng et al, 1994) did not yield a positive identification for the 18 kDa band.

**Figure 3:**
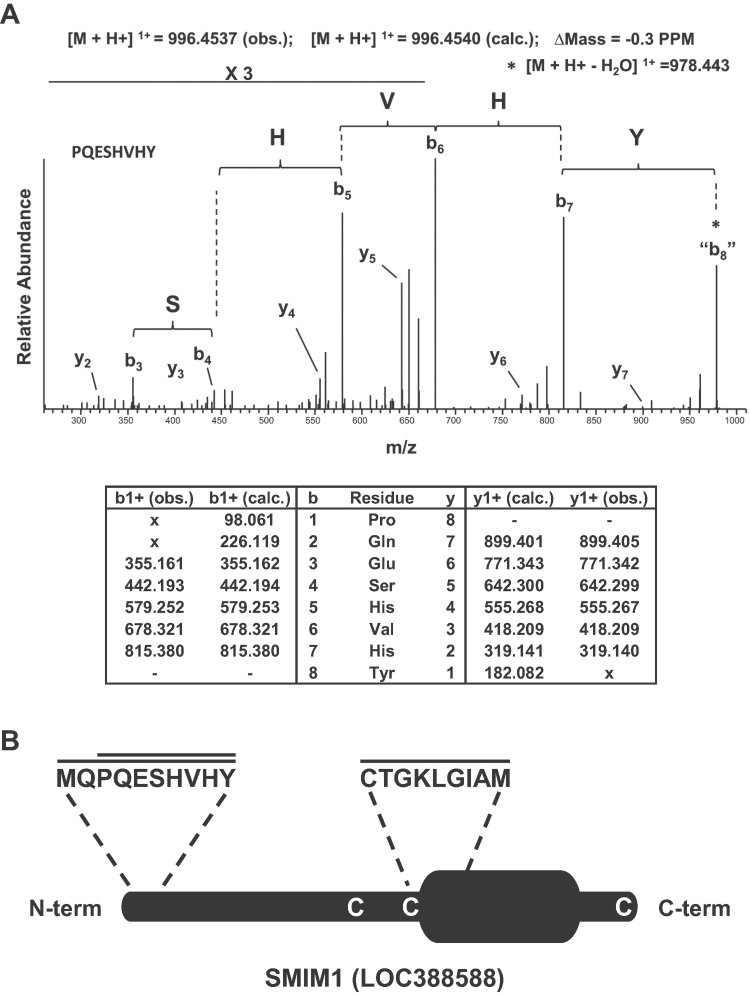
Mass spectrometry-based identification of the Vel antigen carrier as SMIM1. A. The top panel shows the high resolution MS/MS spectrum acquired in the orbitrap mass spectrometer corresponding to the SMIM1 peptide PQESHVHY that was annotated following *de novo* peptide sequencing as described in the Methods Section. The bottom panel provides the corresponding calculated (calc.) and observed (obs.) *m*/*z* values of the singly charged y- and b-type ions. B. Schematic representation of the SMIM1 protein showing the predicted transmembrane domain, the peptides that were identified by mass spectrometry, and the three cysteine residues potentially involved in dimer formation.

Given the relatively small size of the protein carrying the Vel antigen, we had anticipated that its identification would ultimately be determined by relatively few chymotryptic peptides. We had therefore collected the mass spectra for both precursor and fragment ions in a high resolution orbitrap mass spectrometer as this would facilitate *de novo* peptide sequencing if standard analysis programs were to fail. We initiated *de novo* peptide sequencing of our mass spectra using the program PepNovo (Frank & Pevzner, 2005) setting the parameters to identify six residue sequence tags. We manually subjected the top 25 PepNovo-identified hexamer tags to a BLAST search (Altschul et al, 1990) and found that 19 of these hexamer tags were consistent with their mapping to autocatalytic chymotryptic peptides (Supporting Information Table S1). However, three of the six remaining hexamer tags (GKLGIA, ESHVHY and PQESHV) were consistent with their mapping to the uncharacterized protein LOC388588 (Supporting Information Table S1). Manual *de novo* extension mapped these three hexamer tags to three individual, distinct peptides of different precursor masses that covered the following amino acids of LOC388588: CTGKLGIAM, PQESHVHY and MQPQESHVHY. Of note, although the hexamer tags ESHVHY and PQESHV overlap, they originated from entirely different tandem mass spectra resulting from chymotryptic digestion yielded both peptides PQESHVHY and MQPQESHVHY. Fig 3A shows the mass spectrum and *de novo* annotations for the peptide PQESHVHY; the exquisite resolution of the orbitrap mass spectrometer gave less than 1 part per million (PPM) mass accuracy for both parent and identified daughter ions.

LOC388588 was initially identified by the MGC Program Team (Strausberg et al, 2002) as a hypothetical protein predicted to be a single-pass membrane protein (Fig 3B). Coincidentally, LOC388588 was renamed SMIM1 for ‘small integral membrane protein 1’ by the HUGO Gene Nomenclature Committee while this manuscript was under preparation. SMIM1 has no predicted signal peptide, which is fully consistent with the identification by mass spectrometry of the peptide MQPQESHVHY that contains the initiating methionine. Also, SMIM1 possesses three cysteines (Fig 3B) that may be involved in dimer formation. Thus SMIM1 appeared as a genuine carrier of the Vel antigen, which was soon confirmed by the following genetic analysis.

### Genetic basis of the Vel− blood type

**Figure 4:**
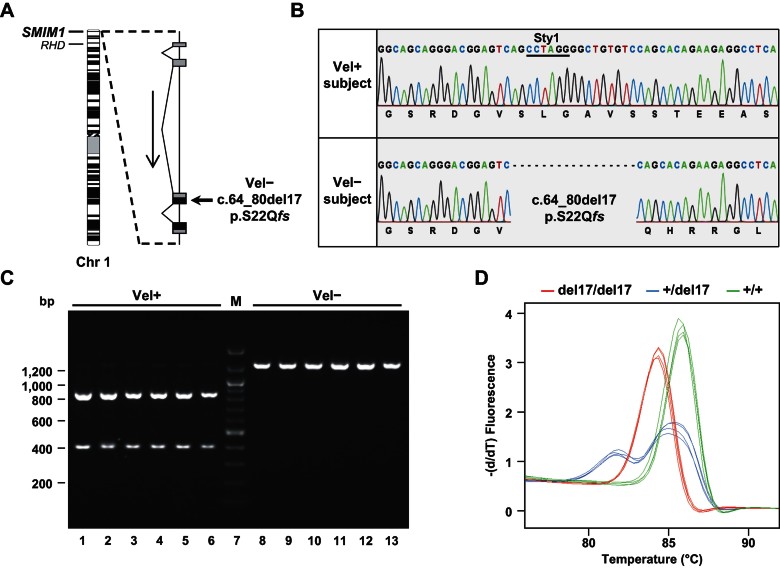
Identification and screening of the 17 nt deletion in *SMIM1* that is responsible for the Vel− phenotype. A. Schematic representation of the *SMIM1* gene showing its location in chromosome 1 (ISCN 550 band ideogram) and its organization in four exons (black represents coding regions, and grey represents untranslated regions). B. Detail of Sanger sequencing of *SMIM1* in a Vel+ subject (top) and a Vel− subject (bottom) showing the homozygous deletion of 17 nt in the *SMIM1* coding sequence (c.64_80del17; pS22Q*fs*) that is responsible for the Vel− blood type. The StyI restriction site on which is based the RFLP analysis is underlined. C. RFLP analysis of the 17 nt deletion in *SMIM1* in six random Vel+ subjects (lanes 1–6) and in six random Vel− subjects (lanes 8–13). D. HRM analysis of the 17 nt deletion in *SMIM1* in three homozygous (red curves), two heterozygous (blue curves) and three wildtype (green curves) individuals, performed in duplicate by two different operators.

The *SMIM1* gene is composed of four exons and is located at the extremity of the short arm of chromosome 1 (1p36.32) in the vicinity of the *RHD* gene, whose disruption is responsible for the well-known RhD− blood type (Fig 4A). Toward the identification of the genetic polymorphism(s) underlying the Vel− blood type, we first amplified and sequenced the *SMIM1* gene in four Vel− subjects and two Vel+ control subjects. We found that all four Vel− subjects were homozygous for a 17 nt deletion in the third exon of *SMIM1* (Fig 4A and B). This deletion introduces a frameshift early in translation (c.64_80del17, p.S22Q*fs*; Fig 4A and B) consistent with the absence of the Vel antigen carrier in Vel− subjects. In contrast, the coding sequence of *SMIM1* was unaltered in Vel+ control subjects (Fig 4B), strongly suggesting that this 17 nt deletion was responsible for the Vel− blood type. This *SMIM1* polymorphism has not yet been reported in the NCBI dbSNP database (build 137).

The homozygous 17 nt deletion that we identified in *SMIM1* from Vel− subjects encompasses a StyI restriction site (Fig 4B), which enabled us to develop a PCR-RFLP analysis to screen for this deletion (Fig 4C). In a cohort of 70 Vel− subjects (64 unrelated and three pairs of siblings), PCR-RFLP analysis revealed that 68 were homozygous for this deletion while two were heterozygous and are discussed in detail below. Sequencing of *SMIM1* from seven random subjects identified in this manner as homozygous for this deletion, as well as from the two subjects identified as heterozygous, confirmed the results of this PCR-RFLP screen. In parallel, PCR-RFLP analysis of 80 Vel+ regular blood donors revealed two individuals heterozygous for this deletion, which is in line with the Hardy–Weinberg expected frequency of the recessive allele responsible for the Vel− blood type whose frequency is 0.0004. Together, these data confirmed that this 17 nt deletion in *SMIM1* is the predominant genetic basis for the Vel− blood type. Hence, we set up a high resolution melting (HRM) analysis of this *SMIM1* deletion in order to facilitate its detection in a clinical context (Fig 4D). Notably, the *Vel* genotyping by HRM analysis is much faster than by RFLP analysis.

During the genotyping of the 70 subjects registered as Vel− two were found to be heterozygous for the 17 nt deletion in *SMIM1* and would be predicted to be Vel+ or perhaps Vel+^w^. Indeed, the genotyping of rare individuals registered as Vel+^w^ revealed that seven out of nine were heterozygous for this deletion. Complete sequencing of *SMIM1* in the two aforementioned subjects confirmed that they were heterozygous for the 17 nt deletion but revealed no compound heterozygosity that could explain the Vel− phenotype of their RBCs. Therefore, we re-analyzed the expression of the Vel antigen on the RBCs of both subjects. For this purpose we used blood samples that were cryopreserved in liquid nitrogen and that could be thawed for haemagglutination or flow cytometry analysis. The RBCs of the first subject appeared to be Vel+^w^, which is fully consistent with her *Vel* genotype, *i.e.* heterozygosity for the 17 nt deletion in *SMIM1*. In contrast, the Vel− phenotype of the second subject's RBCs was confirmed. However, it is important to note that this Vel− subject was 80 years old when identified as Vel− after having developed an anti-Vel. The advanced age of this subject may explain why her RBCs did not express the Vel antigen anymore, as is often observed for the JMH blood group antigen in elderly people (Daniels, 2002).

### Genetic linkage suggests a common origin for the Vel− blood type

The analysis of our cohort of Vel− subjects indicated that the 17 nt deletion in *SMIM1* was the predominant cause of the inherited Vel− phenotype. Therefore, we asked if the prevalence of this null allele of *SMIM1* was due to it having a common origin. If the 17 nt deletion originated from a common ancestral allele of *SMIM1*, we would expect its strong linkage to another nearby genetic landmark. Interestingly, the 11 Vel− subjects homozygous for the 17 nt deletion, whom we sequenced, were also homozygous for the minor allele of the SNP rs71634364 that is located 240 bp downstream (frequency 0.0636). To screen for a possible linkage between these two genetic variations on a larger scale, we set up a PCR-RFLP analysis of rs71634364 (a HinfI restriction site is absent in the minor allele of rs71634364). We conducted this analysis on our cohort of Vel− subjects and found that 67 out of 68 Vel− subjects who were homozygous for the 17 nt deletion were also homozygous for the minor allele of rs71634364. These results indicated a strong linkage between the 17 nt deletion and rs71634364, consistent with the hypothesis of a common origin for the 17 nt deletion in *SMIM1*, and consequently for the Vel− blood type.

### Exogenous expression of *SMIM1* generates the Vel antigen

**Figure 5:**
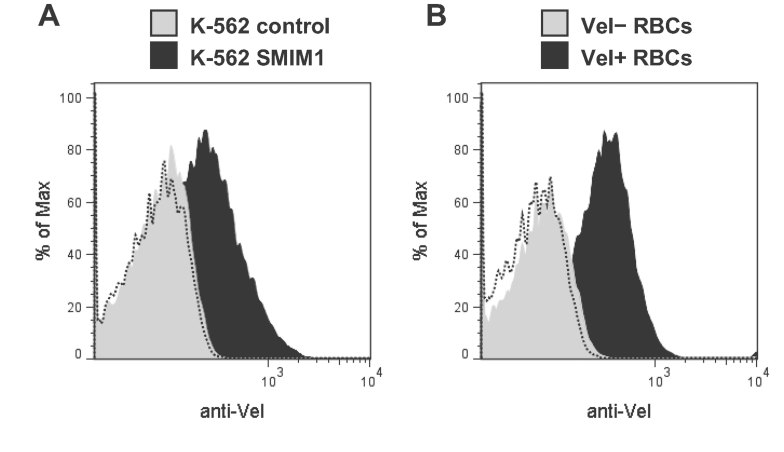
Cell-surface expression of the Vel antigen in *SMIM1*-transfected K-562 cells compared with RBCs. A. Flow cytometry analysis of native K-562 cells stably transfected with a *SMIM1* expression construct (black profile) or with the corresponding empty vector (grey profile) that were labelled with an anti-Vel. The dashed profile corresponds to K-562 SMIM1 cells incubated with only the secondary antibody. B. Flow cytometry analysis of native RBCs taken from a Vel+ subject (black profile) or a Vel− subject (grey profile) labelled with an anti-Vel. The dashed profile corresponds to Vel+ RBCs incubated with only the secondary antibody.

Finally, we assessed whether expression of *SMIM1* in an exogenous system would generate the Vel antigen. If this were possible it would not only verify the role of *SMIM1* in the Vel− blood type, but it could establish a positive control cell line that would be a valuable tool to identify anti-Vel. We cloned the cDNA of *SMIM1* into an episomal expression vector and we transfected this construct into K-562 erythroleukaemia cells. Flow cytometry analysis of K-562 cells stably transfected with the *SMIM1* cDNA showed cell-surface expression of the Vel antigen (Fig 5A, black profile). In contrast, K-562 cells stably transfected with the corresponding empty vector did not (Fig 5A, grey profile), confirming that expression of the Vel antigen strictly depends on expression of *SMIM1*. Notably, exogenous expression of the Vel antigen in K-562 cells mirrored endogenous expression of the Vel antigen in RBCs (compare Fig 5A and B).

## DISCUSSION

Using a biochemical approach complemented with a genetic study, we have identified the molecular basis of the long sought-after Vel blood group antigen. We found the Vel antigen to be carried by SMIM1 on RBCs. Furthermore, we found in a cohort of 70 Vel− subjects that the inherited absence of the Vel antigen (*i.e.* the Vel− blood type) shows almost complete correspondence to the homozygosity of a 17 nt deletion that disrupts the *SMIM1* coding sequence. Intriguingly, we found this 17 nt deletion to be linked to a low-frequency SNP in *SMIM1* suggestive of a common origin, although population analyses beyond the scope of this study will be required to reveal its likely geo-anthropological or possible biophysical root.

Even though almost all the Vel− persons reported so far were of European descent including our cohort of 70 Vel− subjects, a few cases among other ethnicities have been reported. Four Thai persons were identified as Vel− in 1967 during an extended phenotyping of 328 blood donors from Bangkok (Chandanayingyong et al, 1967). It would be interesting to determine whether or not the Vel− phenotype in persons of Thai descent results from the same *SMIM1* disruption. Two out of 160 Chilcotin First Nations people (British Columbia, Canada) were also reported to be Vel− but this result should be interpreted with caution as the authors of this ethnologically oriented study did not mention their source of anti-Vel (Alfred et al, 1970). As a matter of fact, a similar study of 133 Penobscot Native Americans (Maine, USA) with a potent anti-Vel revealed no Vel− subjects ‘though two siblings were such weak positives that they were at first thought to be negative’ (Allen & Corcoran, 1960).

While we cannot formally conclude that the 17 nt deletion in *SMIM1* is the only genetic basis of the Vel− blood type, there is currently no evidence for other genetic origins of the Vel− blood type. The discovery of this 17 nt deletion in *SMIM1* as the major, and maybe the only, cause of the Vel− blood type will have an immediate impact in transfusion medicine. Indeed, the Vel− blood type can now be predicted with high confidence by analyzing genomic DNA. This will reduce the reliance of reference laboratories on the notoriously recalcitrant anti-Vel antibody. Depending on the laboratory setting, we show herein two possible techniques (HRM and RFLP) for rapid assessment of the *Vel* genotype. Of note, the HRM analysis is better suited for routine laboratories and can be performed in less than 2 h, which is rapid enough to be influential in emergency situations. Additionally, *Vel* genotyping could now be integrated into the current blood group genotyping platforms. Large-scale *Vel* genotyping of blood donors should ultimately solve the Vel− blood supply challenge (Jungbauer, 2009; Veldhuisen et al, 2009).

Previous studies aiming at determining the genetic basis of the Vel− blood type, including a recent exome sequencing study (European Genome-phenome Archive, Study ID EGAS169), were unsuccessful. At first glance this would be unexpected given that we found that a single 17 nt deletion in *SMIM1* is the predominant cause of the Vel− blood type. However, until recently SMIM1 was annotated only as a hypothetical protein (*i.e.* LOC388588) and thus *SMIM1* may not have been present in exome arrays. Although paved with obstacles, our biochemical purification of a protein unique to Vel+ individuals eventually provided the needed handle to wrest the problem of the Vel− blood type. These obstacles were overcome first thanks to a potent anti-Vel, which we succeeded to use for Western blot analysis. All our attempts with other anti-Vel were unsuccessful. Secondly, the fortuitous observation that SMIM1 runs at distinctly different molecular weights under reducing and non-reducing conditions provided the basis for a unique purification strategy. Several attributes of purified SMIM1, however, made its identification refractory to standard mass spectrometry workflows: (i) SMIM1 was nearly untouched by trypsin, the workhorse enzyme in bottom-up proteomics; (ii) SMIM1 is small and even the few possible chymotryptic peptides were not identified by a standard proteomic search algorithm in spite of the good fortune that SMIM1 (*i.e.* LOC388588) was indeed in the proteomic database that we searched; (iii) SMIM1 would not have been identified without the non-standard acquisition of high resolution MS/MS data and manual *de novo* peptide sequencing. Our mass spectrometry approach can therefore serve as a general guide to the identification of proteins resisting discovery by standard proteomics platforms.

However, while troubleshooting these difficulties, important biophysical information about the previously uncharacterized SMIM1 and the nature of the Vel antigen were uncovered. First, we now know that SMIM1 can form molecular complexes (likely homodimers) via disulphide bonds. Second, SMIM1 assumes a conformation that is largely resistant to trypsin in spite of its containing several arginine and lysine residues. Third, the positive results of the anti-Vel Western blot under denaturing and reducing conditions divulge that the epitope recognized by the anti-Vel does not require SMIM1 to be in a native conformation or a molecular complex. Fourth, the SMIM1 protein is not required for normal development and physiology given that individuals lacking SMIM1 (*i.e.* having the Vel− blood type) have no known clinical manifestations.

While the exact epitope recognized by anti-Vel remains to be defined, the small size, as well as the predicted structure of SMIM1 with a single transmembrane domain, limits the number of potential epitopes. Toward this goal, it would be important to determine whether SMIM1 is a type I or II membrane protein, *i.e.* whether its N-terminus is extra- or intracellular. Of note, most type I membrane proteins have a N-terminal cleavable signal peptide in contrast with type II membrane proteins, whose transmembrane domain functions as a (non-cleavable) signal peptide and as a membrane anchor. Our mass spectrometry identification of extreme N-terminal SMIM1 amino acids is consistent with SMIM1 being a type II membrane without a N-terminal cleavable signal peptide. One may also note that SMIM1 does not migrate at its theoretical molecular weight (8.749 kDa) in SDS–PAGE even under reducing conditions. This may reflect the presence of post-translation modifications including glycosylations or phosphorylations. Nevertheless, one should not neglect that membrane proteins often deviate from their theoretical molecular weight in SDS–PAGE as hydrophobic transmembrane domains bind less SDS (Rath et al, 2009).

In conclusion, we have identified SMIM1 to be the protein behind the mystery of the Vel antigen and have therefore uncovered the basis for a novel human blood group system. We anticipate that this knowledge and the assays herein described to detect deletions in *SMIM1* will reduce the burden and anxiety of patients and health care professionals when confronted with the Vel− blood type.

## MATERIALS AND METHODS

### Blood samples

This study was conducted according to the ethical standards of the National Institute of Blood Transfusion (INTS, Paris, France). The analysis of *SMIM1* in Vel− subjects was conducted on historical blood samples that had been cryopreserved in the rare blood collection of the National Reference Center for Blood Groups (CNRGS, Paris, France) in order to establish the Vel− blood type of other subjects, and ultimately to identify the genetic basis of Vel− blood type. The analysis of *SMIM1* in control Vel+ subjects was conducted on gDNA samples that had been extracted previously (Kappler-Gratias et al, 2011). Fresh blood samples were obtained after informed consent.

### Antibody purification

Anti-Vel was purified by adsorption of a serum sample of Mrs. ‘Jem’ (A blood type), which had been donated in 1995 and cryopreserved in the serum collection of the National Reference Center for Blood Groups (CNRGS), onto a pool of three papain-treated erythrocytes with the O blood type, and eluted by low pH treatment with Gamma ELU-KIT II (Immucor Gamma) according to the manufacturer's instructions. The Vel specificity of the eluate as well as the absence of contaminating anti-B was checked.

### Western blot analysis

Lysates of erythrocyte membranes were prepared from blood samples taken on EDTA. Erythrocyte membranes were prepared at 0–4°C by hypotonic lysis with 5P8 buffer (5 mM Na_2_HPO_4_ pH 8.0 and 350 µM EDTA pH 8.0) supplemented with 1 mM AEBSF (Uptima), and solubilized at 100°C for 5 min in 10 mM Tris–HCl pH 6.8, 1.5% SDS and 6% glycerol. Twenty micrograms of lysates, reduced with 100 mM DTT or left untreated, were resolved by Tris–glycine SDS–PAGE and transferred to PolyScreen PVDF Transfer Membrane (Perkin Elmer) by submarine transfer. The Mark12 Unstained Standard (Invitrogen) was used as reference of molecular weights. PVDF membranes were blocked for 16 h at 4°C in low-ionic strength BFI buffer (240 mM glycine, 30 mM NaCl, 1.5 mM NaH_2_PO_4_ and 1.5 mM Na_2_HPO_4_) supplemented with 3% BSA (Sigma–Aldrich; cat. A3174) and 0.05% Tween 20, and incubated with anti-Vel eluate (1:10) for 2 h 45 min at 21°C. PVDF membranes were then blocked 1 h at 21°C in PBS (130 mM NaCl, 5.1 mM Na_2_HPO_4_ and 1.5 mM KH_2_PO_4_) supplemented with 5% non-fat dry milk (Régilait) and 0.05% Tween 20, and incubated with anti-human IgGAM horseradish-peroxidase-linked goat antibody (1:5000; P.A.R.I.S Biotech) for 45 min at 21°C. Horseradish-peroxidase activity was revealed with the Amersham ECL Plus Western Blotting Detection System (GE Healthcare) and Kodak BioMax MR films (Eastman Kodak Company). After treatment with 0.02% azide, PVDF membranes were reprobed with a historical, home-made and affinity-purified rabbit anti-7.2b.

### Protein purification

Erythrocyte membrane extracts were prepared from blood units taken on citrate from regular blood donors. Erythrocyte membranes were prepared at 0–4°C by hypotonic lysis with 5P8 buffer supplemented with 1 mM AEBSF, stripped by incubation with 10 mM NaOH and finally solubilized with an equal volume of 4× LDS Sample Buffer (Invitrogen). These lysates were resolved by Tris–glycine 11% SDS–PAGE, and the material of approximately 32 kDa was eluted with Model 422 Electro-Eluter and Membrane Caps with a molecular weight cut-off of 12–15 kDa (BioRad) according to the manufacturer's instructions. After reduction with 100 mM DTT, this electro-eluted material was then resolved by Tris–glycine 13% SDS–PAGE and the material of approximately 18 kDa, which corresponds to the Vel antigen carrier protein, was subjected to in-gel digestion or eluted with Model 422 Electro-Eluter and Membrane Caps with a molecular weight cut-off of 3.5 kDa (BioRad) according to the manufacturer's instructions.

### Mass spectrometry and data analysis

After washing the gel pieces with water and dehydrating them with 100% acetonitrile they were subjected to in-gel reduction with 25 mM DTT, followed by dehydration with 100% acetonitrile and then in-gel alkylation with 50 mM iodoacetamide in 50 mM ammonium bicarbonate for 30 min in the dark. After two rounds of dehydration with acetonitrile and rinsing with water, the gel pieces were subjected to in-gel digestion with either trypsin or chymotrypsin. These procedures were performed as described previously (Ballif et al, 2006) except that digestion with chymotrypsin (12.5 ng/µl) occurred at room temperature for 2 h. Extracted and dried peptides were subjected to liquid chromatography tandem mass spectrometry (LC–MS/MS) analysis using a linear ion trap-orbitrap hybrid mass spectrometer (LTQ-orbitrap, Thermo-Electron, San Jose, CA) set up as described previously (Haas et al, 2006; Roskens et al, 2010). For tryptic peptides, precursor scans (360–1600 *m*/*z*) were conducted in the orbitrap followed by 10 data-dependent MS/MS spectra in the linear ion trap. Chymotryptic peptides were analyzed similarly except that all data were acquired in the orbitrap. Dynamic exclusion was enabled with a repeat count of three and a repeat cycle of 180 s. Lock mass was enabled and set to calibrate on the mass of a polydimethylcyclosiloxane ion ([(Si(CH_3_)_2_O)_5_ + H^+^]^+^, *m*/*z* = 371.10120). SEQUEST searches (Eng et al, 1994) were conducted as described previously (Ballif et al, 2008) except that dynamic carbamidomethylation of cysteine was permitted and for chymotryptic searches no specific enzyme specificity was required. *De novo* sequencing was initiated with the program Pepnovo (Frank & Pevzner, 2005) by loading RAW files to the University of California at San Diego's Center for Computational Mass Spectrometry Pepnovo server where the following selections were made: high accuracy instrument, methionine oxidation, cysteine carbamidomethylation, no protease, parent ion tolerance of 0.01, MS/MS ion tolerance of 0.01 and a hexamer mass tag was required. The top 25 Pepnovo-reported hexamers were subjected to a BLAST search (Altschul et al, 1990). For peptides mapping to SMIM1 the corresponding MS/MS spectra were manually examined for the presence of b- and y-type ions that would extend the sequence in a manner consistent with the mass of the chymotryptic precursor, the sequence of SMIM1, and the low mass error tolerance as performed previously (Roskens et al, 2010).

### Genomic DNA sequencing and data analysis

The full-length *SMIM1* gene was amplified with primers LOC388588-1 and LOC388588-6 (Supporting Information Table S2) from genomic DNA isolated from whole blood using the Wizard Genomic DNA Purification Kit (Promega). The corresponding PCR product was purified with SureClean Plus (Bioline) and sequenced by ABI BigDye terminator chemistry (GATC Biotech) with primers LOC388588-7 and LOC388588-4, and LOC388588-5 in case of heterozygosity for the deletion c.64_80del17. Of note, the two coding exons of *SMIM1* can be amplified with primers LOC388588-3 and LOC388588-6, and sequenced with LOC388588-4 and LOC388588-5. Sequencing data were analyzed with DNA Workbench software (CLC bio) and Mutation Surveyor software (SoftGenetics). Mutation numbering relates to the reference sequence of *SMIM1* mRNA (NM_001163724.1) with the +1 position referring to the first nucleotide of the initiation codon. The sequence of the *SMIM1* gene from individuals with the Vel− blood type has been submitted to the Genbank database and assigned the accession number KC751412.

**The paper explained**

**PROBLEM**

Intense efforts in clinical research, as well as in basic research, have uncovered the molecular basis of most blood groups, yielding important improvements in transfusion medicine and obstetrics. However, the molecular basis of a few blood groups have stubbornly eluded discovery in spite of their clinical significance. Such has been the case for the Vel− blood group that was recognized in 1952 and that is regularly responsible for life-threatening transfusion accidents. More than 200,000 persons in Europe, and as many in North America, are estimated to have the Vel− blood group.

**RESULTS**

In this manuscript, we describe the molecular basis underlying the Vel− blood group, whose characterization required original approaches. The protein carrying the Vel blood group antigen was purified from red blood cell membranes on the basis of its unique electrophoretic migration. While its identification was refractory to standard workflows, cutting-edge proteomics technologies identified the protein carrying the Vel blood group antigen to be small integral membrane protein 1 (SMIM1), a previously uncharacterized single-pass membrane protein. Cellular analyses as well as a genetic analysis of a cohort of 70 Vel− individuals showed that disruption of *SMIM1* is the underlying genetic cause for the Vel− blood group. Altogether, these data establish Vel as a novel blood group system. Finally, and in order to translate this discovery into actual treatment, we have developed two rapid analyses for genotyping the Vel− blood group.

**IMPACT**

Our results provide tools of immediate assistance to clinicians involved in the care of Vel− patients. Through a simple genetic test that circumvents the inherent limitations of serological blood typing, the Vel− blood group can now be identified quickly and efficiently, thereby improving its clinical management. Furthermore, the mechanism by which our methodology links phenotype to genotype will be of broad interest to the scientific community given it outlines approaches that fall outside standard platforms.

### Genotyping

Genomic DNA extraction from cryopreserved Vel− blood samples was performed with a QIAsymphony SP instrument and a QIAsymphony DNA Midi Kit (Qiagen) according to the manufacturer's instructions. Genomic DNA from control Vel+ regular blood donors had been extracted similarly (Kappler-Gratias et al, 2011). DNA concentration was measured with a NanoDrop 2000c spectrophometer (Thermo Scientific).

For the *Vel* genotyping by RFLP analysis, a fragment encompassing exons 3 and 4 of *SMIM1* was amplified from genomic DNA by PCR with primers LOC388588-3 and LOC388588-5 (Supporting Information Table S2), which were synthesized by Eurofins MWG Operon. The PCR amplification was made in a 2720 Thermal Cycler (Applied Biosystems) in 11 µl reactions containing 1.5 µl genomic DNA (10–200 ng/µl), 0.2 µl each primer (10 µM), 0.1 µl Advantage GC Genomic LA Polymerase Mix (Clontech Laboratories) and 5 µl FailSafe PCR 2X Premix D (Epicentre Biotechnologies). The amplification program consisted of an initial step at 94°C for 2 min, 35 cycles at 94°C for 30 s, 58°C for 30 s and 72°C for 90 s, and a final extension step at 72°C for 3 min. While this amplification was successful for genomic DNA samples as low as 1 ng/µl, a second amplification was sometimes necessary for samples <10 ng/µl. RFLP analysis was carried out with 2 µl of PCR reaction in a 10 µl reaction containing 3 U of StyI restriction endonuclease (New England Biolabs) in 1× NEBuffer 3 at 37°C for 2 h, and revealed by 1.2% agarose gel electrophoresis in 1× TBE buffer.

For the *Vel* genotyping by HRM analysis, an internal fragment of exon 3 of *SMIM1* was amplified from genomic DNA by PCR with primers LOC388588-qPCR1 and LOC388588-qPCR4 (Supporting Information Table S2), which were synthesized by Eurogentec. The PCR amplification and the HRM analysis were performed in a single run in a LightCycler 480 System (Roche Diagnostics) in 20 µl reactions containing 20 ng of genomic DNA, 70 nM of each primer in 1× Absolute Blue SYBR Green Mix (Thermo Scientific). The amplification program consisted of 40 cycles at 95°C for 10 s, 60°C for 45 s and 72°C for 10 s after an activation step at 95°C for 15 min. The melting program consisted of a gradient from 55 to 95°C with an increment of 0.11°C/s, after a denaturation step at 95°C for 20 s and an annealing step at 55°C for 60 s. HRM curve analysis was performed using the LightCycler 480 Software version 1.5.

### Plasmid construction

The coding sequence of *SMIM1* cDNA was amplified with primers LOC388588-8 and LOC388588-9 (Supporting Information Table S2) from a Human Bone Marrow Marathon-Ready cDNA library (Clontech), cloned into pCR4Blunt-TOPO vector (Invitrogen), sequence-verified (identical to NM_001163724.1) and subcloned as a NotI/SbfI fragment into the pCEP5 episomal vector, which is a pCEP4 vector (Invitrogen) with a modified polylinker. The sequence file of the pCEP5-SMIM1 plasmid can be obtained upon request.

### Cell culture and transfection

Human K-562 cells were grown in Iscove's modified Dulbecco's medium (IMDM + GlutaMAX-I, Gibco) supplemented with 10% foetal bovine serum (Pan-Biotech) and 0.5× antibiotic-antimycotic solution (Gibco) at 37°C under a humidified atmosphere containing 5% CO_2_. Cells were periodically tested for mycoplasma contamination using a home-made PCR assay. To obtain K-562 transfectants, 10^6^ K-562 cells were first transfected with 2 µg of the pCEP5-SMIM1 plasmid by nucleofection using the Nucleofector II device and the Cell Line Nucleofector Kit V (Amaxa) according to the manufacturer's protocol (program T-016). Stable K-562 transfectants were obtained after 7–8 days of selection with hygromycin B (0.8 mg/ml, Invitrogen).

### Flow cytometry analysis

Thawed RBCs or fresh K-562 cells were washed in Dulbecco's phosphate-buffered saline solution (DPBS, Gibco) and then resuspended in low-ionic strength BFI buffer supplemented with 0.15% BSA, and incubated with anti-Vel eluate (1:2). Anti-Vel labelling was revealed with goat F(ab')_2_ anti-human IgG(H + L)-PE (1:100; Beckman Coulter) and immediately analyzed with a FACSCantoII flow cytometer (BD Bioscience) equipped with FACSDiva software (v. 6.1.2; BD Bioscience). Ten thousand RBCs or viable K-562 cells, gated on forward scatter (FSC) *versus* side scatter (SSC), were collected for each sample. Data were analyzed with FlowJo software (v. 7.6.5; TreeStar).

## Author contributions

BAB solved the identity of the Vel carrier protein by mass-spectrometry analysis and wrote the manuscript. VH performed the large-scale purification of the Vel carrier protein, genomic DNA sequencing and flow cytometry analysis. TP provided immunohaematological information and the 70 cryopreserved Vel− blood samples. CM purified the anti-Vel. CS prepared RBC membrane extracts and performed silver staining analysis. NL performed anti-Vel Western blot analysis. SB performed genomic DNA extraction from cryopreserved Vel− blood samples. MLG designed and performed the HRM analysis. JPC continuously supported the project. LA conceived and supervised the study, set up the purification of the Vel carrier protein, designed and performed the RFLP analysis, made the DNA constructs, made the figures and wrote the manuscript. The manuscript was edited by MLG, TP and JPC and was approved by all authors before submission.

## Notes

As a service to our authors and readers, this journal provides supporting information supplied by the authors. Such materials are peer reviewed and may be re-organized for online delivery, but are not copy-edited or typeset. Technical support issues arising from supporting information (other than missing files) should be addressed to the authors.
