# Supplementary material for: Disruption of SMIM1 causes the Vel− blood type
Source: EMBO Mol Med. 2013 Apr 15;5(5):751–61. doi: 10.1002/emmm.201302466 (PMC3662317; doi:10.1002/emmm.201302466)
Supplement: Supplementary file 1 [file emmm0005-0751-sd1.pdf]

EMBO Molecular Medicine

Disruption of SMIM1 causes the Vel blood type

Bryan A. Ballif, Virginie Helias, Thierry Peyrard, Cécile Menanteau, Carole Saison, Nicole Lucien, Sébastien Bourgouin, Maude Le Gall, Jean-Pierre Cartron and Lionel Arnaud

Corresponding author: Lionel Arnaud, National Institute of Blood Transfusion

Review timeline:

Submission date:	07 January 2013
Editorial Decision:	14 February 2013
Revision received:	05 March 2013
Additional Author Correspondence:	08 March 2013
Accepted	10 March 2013

Transaction Report:

(Note: With the exception of the correction of typographical or spelling errors that could be a source of ambiguity, letters and reports are not edited. The original formatting of letters and referee reports may not be reflected in this compilation.)

Editor: Natascha Bushati / Céline Carret

1st Editorial Decision 14 February 2013

Thank you for the submission of your manuscript to EMBO Molecular Medicine. We are very sorry that it has taken so long to get back to you on your manuscript.

While reviewers 1 and 2 delivered their evaluations in a timely manner, we have not yet received the third reviewer's input. As a further delay cannot be justified, I have decided to proceed based on these evaluations.

As you will see from the comments below, both reviewers found your manuscript of interest. Nevertheless, they also have some concerns that I would like you to convincingly address in a revised version of your manuscript. Please be aware that depending on the nature of the revisions, we may decide to send back the manuscript to the referees for another round of review.

Please note that it is EMBO Molecular Medicine policy to allow a single round of revision only and that, therefore, acceptance or rejection of the manuscript will depend on the completeness of your responses included in the next, final version of the manuscript.

As you know, EMBO Molecular Medicine has a "scooping protection" policy, whereby similar findings that are published by others during review or revision are not a criterion for rejection. However, I do ask you to get in touch with us after three months if you have not completed your revision, to update us on the status. Please also contact us as soon as possible if similar work is published elsewhere.

I look forward to receiving your revised manuscript.

***** Reviewer's comments *****

Referee #1 (Comments on Novelty/Model System):

This is an important contribution to our knowledge of blood group antigens and one of the last antigens to be characterized. The Vel antigen is rare but because it can cause strong hemolytic reactions understanding the molecular genetics and biochemistry is important for transfusion safety and development of typing reagents and methods.

Referee #1 (General Remarks):

This manuscript describes the molecular genetic basis of the Vel blood group antigen - a rare and important antigen that can cause severe hemolytic reactions, which has been a puzzle for over 50 years. The authors use a potent anti-Vel antiserum that Western blots to isolate the 32/18 kd protein and partially sequence it by de novo mass spec sequencing of trypsin in-gel digests. The sequences identified matched a predicted small protein with a single hydrophobic sequence in the C-terminal region, and sequencing of the gene (SM1M1) in Vel positive and Vel negative individuals demonstrated a 17 nt deletion in Vel negatives, confirming the identity of this protein as representing the Vel antigen. This was further substantiated by expression of the gene in K562 cells. The experimental design and results are clear and conclusive and the manuscript is well written. The molecular genetic basis of the Vel blood group antigen is important for the transfusion field and will have wider general interest.

The first sentence in the abstract claims that the report includes the biochemical basis of the Vel antigen (not in the title!), but this may be argued as the antigenic epitope is not clarified per se. This reviewer would suggest that a discussion of the potential epitope is included. This could include a discussion of the unusual predicted topology of the protein without N-terminal signal sequence (does the C-terminal hydrophobic domain serve as non-cleavable signal peptide?) and a correlation of predicted mass and the estimated mass. It appears that the protein is only 78 amino acids (include Fig of sequence and predicted topology?), which is difficult to envision running at 18/32 kd as mono/dimer?

Referee #2 (Comments on Novelty/Model System):

The study required a high level of technical expertise in order to isolate the protein but the metods required.
Identification of Vel protein and gene, and the molecular basis of the Vel-negative phenotype.
This information is valuable for confirming Vel-negative phenotype and for screeing for Vel-negative donors. As extended blood group phenotyping of blood donors by molecular methods is starting to be introduced widely, this will provide a useful addition.
I must declare that my expertise lies in blood group genetics and I am not qualified to comment on the biochemical analyses employed.

Referee #2 (General Remarks):

This is a well-written and interesting manuscript describing the identification of the gene encoding the Vel blood group antigen and the molecular background of the rare Vel-negative phenotype. I only have a few minor comments.

Title
VEL is not a novel blood group system. Although the findings reported in this paper are novel, Vel is a blood group antigen that has been known for decades. A VEL system may be created by the International Society of Blood Transfusion as a result of these findings, but this is not mentioned in the paper.

Abstract
Lines 2. Although anti-Vel has caused severe hemolytic transfusion reaction, it is a gross

exaggeration to say that it 'regularly' causes these.
Line 8. 'Strikingly' is not required.

Introduction
Page 3, bottom line.
I am not sure what is meant by 'notorious obstinance' (and I don't think that obstinance is a word). This should be changed.
The term 'high-titer, low avidity' is outdated and unhelpful. In addition, even when the term was used, it was not usually applied to anti-Vel. The main reason why many examples of anti-Vel are difficult to detect is that they are IgM and only reactive in an antiglobulin test when a source of complement is present.

Results
Page 8, end of first paragraph. This is misleading. I know of no evidence that Vel expression is lost with age. The comparison with JMH is irrelevant.
Page 8, second paragraph. The authors claim that the 17 nt deletion is the predominant cause of Vel-negative and suggest that this demonstrates a founder effect. However, they do not mention the ethnicity of the Vel-negative individuals tested. Vel-negative phenotype has been found in ethnic groups other than Caucasians, for example Thais and native Americans, and they may have other mutations. If the authors are unable to test Vel-negatives from other ethnic groups, they should state the ethnicity of those tested. If they do not know and are unable to find out, they should discuss the issue.

References
Ballif et al 2006. Is '3rd and' an error?

Legend to Fig. 2, one up from bottom. I suggest 'hash symbols' in place of 'number signs'.

1st Revision - authors' response 05 March 2013

***** Point-by-point response to *Reviewer's comments* *****

Referee #1 (Comments on Novelty/Model System):

This is an important contribution to our knowledge of blood group antigens and one of the last antigens to be characterized. The Vel antigen is rare but because it can cause strong hemolytic reactions understanding the molecular genetics and biochemistry is important for transfusion safety and development of typing reagents and methods.

Referee #1 (General Remarks):

This manuscript describes the molecular genetic basis of the Vel blood group antigen – a rare and important antigen that can cause severe hemolytic reactions, which has been a puzzle for over 50 years. The authors use a potent anti-Vel antiserum that Western blots to isolate the 32/18 kd protein and partially sequence it by de novo mass spec sequencing of [chymo-] *trypsin in-gel digests. The sequences identified matched a predicted small protein with a single hydrophobic sequence in the C-terminal region, and sequencing of the gene (SM1M1) in Vel positive and Vel negative individuals demonstrated a 17 nt deletion in Vel negatives, confirming the identity of this protein as representing the Vel antigen. This was further substantiated by expression of the gene in K562 cells.*

The experimental design and results are clear and conclusive and the manuscript is well written. The molecular genetic basis of the Vel blood group antigen is important for the transfusion field and will have wider general interest.

The first sentence in the abstract claims that the report includes the biochemical basis of the Vel antigen (not in the title!), but this may be argued as the antigenic epitope is not clarified per se. This reviewer would suggest that a discussion of the potential epitope is included. This could include a discussion of the unusual predicted topology of the protein without N-terminal signal sequence

(does the C-terminal hydrophobic domain serve as non-cleavable signal peptide?) and a correlation of predicted mass and the estimated mass. It appears that the protein is only 78 amino acids (include Fig of sequence and predicted topology?), which is difficult to envision running at 18/32 kd as mono/dimer?

As suggested by this reviewer, we have included in the Discussion section the hereafter paragraph to discuss the potential epitopes of anti-Vel in the SMIM1 protein, the absence of a signal peptide for this single-pass membrane protein, as well as the difference between its theoretical molecular weight and its observed molecular weight in SDS-PAGE.

'While the exact epitope recognized by anti-Vel remains to be defined, the small size, as well as the predicted structure of SMIM1 with a single transmembrane domain, limits the number of potential epitopes. Toward this goal, it would be important to determine whether SMIM1 is a type I or II membrane protein, i.e., whether its N-terminus is extra- or intracellular. Of note, most type I membrane proteins have a N-terminal cleavable signal peptide in contrast with type II membrane proteins, whose transmembrane domain functions as a (non-cleavable) signal peptide and as a membrane anchor. Our mass spectrometry identification of extreme N-terminal SMIM1 amino acids is consistent with SMIM1 being a type II membrane without a N-terminal cleavable signal peptide. One may also note that SMIM1 does not migrate at its theoretical molecular weight (8.7 kDa) in SDS-PAGE even under reducing conditions. This may reflect the presence of post-translation modifications including glycosylations or phosphorylations. Nevertheless, one should not neglect that membrane proteins often deviate from their theoretical molecular weight in SDS-PAGE as hydrophobic transmembrane domains bind less SDS (Rath et al, 2009).'

Referee #2 (Comments on Novelty/Model System):

The study required a high level of technical expertise in order to isolate the protein but the methods required.
Identification of Vel protein and gene, and the molecular basis of the Vel-negative phenotype.
This information is valuable for confirming Vel-negative phenotype and for screening for Vel-negative donors.
As extended blood group phenotyping of blood donors by molecular methods is starting to be introduced widely, this will provide a useful addition.
I must declare that my expertise lies in blood group genetics and I am not qualified to comment on the biochemical analyses employed.

Referee #2 (General Remarks):

This is a well-written and interesting manuscript describing the identification of the gene encoding the Vel blood group antigen and the molecular background of the rare Vel-negative phenotype.

I only have a few minor comments.

Title
VEL is not a novel blood group system. Although the findings reported in this paper are novel, Vel is a blood group antigen that has been known for decades. A VEL system may be created by the International Society of Blood Transfusion as a result of these findings, but this is not mentioned in the paper.

We totally agree that the Vel blood group antigen has been known for decades, as clearly stated in our manuscript ('The existence of the Vel antigen was recognized in 1952 by Sussman and Miller who found an alloantibody with a novel specificity in the serum of Mrs. "Vel" who suffered an acute HTR (Sussman & Miller, 1952).'). Our manuscript reports the identity of the protein carrying the Vel antigen, the identity of the gene encoding the Vel antigen, and the identity of a predominant mutation responsible for the Vel− blood type, hence specifying a novel blood group system according to the International Society of Blood Transfusion (ISBT). Nevertheless, this reviewer is right when saying that VEL is not (yet) a novel blood group system until the ISBT officially creates

it as a result of our findings. This should be done at the next International Congress of the ISBT in June 2014 (Seoul, South Korea).
Therefore, we have changed the title of our manuscript and it now reads, "Disruption of *SMIM1* causes the Vel− blood type".

Abstract
Lines 2. Although anti-Vel has caused severe hemolytic transfusion reaction, it is a gross exaggeration to say that it 'regularly' causes these.

We respectfully disagree with this comment. When only considering France, there are two life-threatening haemolytic transfusion reactions (HTR) caused by anti-Vel per year on average. Of note, we only mention here registered transfusion accidents that are unambiguously associated with anti-Vel. The annual number of severe transfusion accidents that are potentially associated with anti-Vel is much higher.

Line 8. 'Strikingly' is not required.

'Strikingly' has been removed, as suggested by this reviewer.

Introduction
Page 3, bottom line.
I am not sure what is meant by 'notorious obstinance' (and I don't think that obstinance is a word). This should be changed.

'Notorious obstinance' has been replaced by 'notorious reluctance' to elegantly express that most immunohematologists think that anti-Vel is a very difficult antibody to work with *in vitro*.

The term 'high-titer, low-avidity' is outdated and unhelpful. In addition, even when the term was used, it was not usually applied to anti-Vel.

We thank the reviewer for this comment but we respectfully disagree. The term 'high-titer, low affinity' (HTLA) is still used extensively, not only in the French National Reference Center for Blood Groups but also in many other European reference laboratories, even though some immunohematologists think that this term is outdated. As a matter of fact, the HTLA characteristics of anti-Vel are mentioned in the Table 30-3 of the reference book 'Applied Blood Group Serology' from Prof Peter D. Issit and Prof David J. Anstee (Fourth Edition, April 1998, Montgomery Scientific Publications).

The main reason why many examples of anti-Vel are difficult to detect is that they are IgM and only reactive in an antiglobulin test when a source of complement is present.

We thank the reviewer for this technical comment.

Page 8, end of first paragraph. This is misleading. I know of no evidence that Vel expression is lost with age. The comparison with JMH is irrelevant.

We thank the reviewer for sharing professional experience. Our comment was made to propose a potential explanation by mentioning that 'The advanced age of this subject may explain why her RBCs did not express the Vel antigen anymore, as is often observed for the JMH blood group antigen in elderly people'. We made the comparison with JMH on purpose because this blood group antigen is the paradigm of expression loss with age. We leave the decision to the editor to keep or remove this sentence.

Page 8, second paragraph. The authors claim that the 17 nt deletion is the predominant cause of Vel-negative and suggest that this demonstrates a founder effect. However, they do not mention the ethnicity of the Vel-negative individuals tested. Vel-negative phenotype has been found in ethnic groups other than Caucasians, for example Thais and native Americans, and they may have other mutations. If the authors are unable to test Vel-negatives from other ethnic groups, they should state

the ethnicity of those tested. If they do not know and are unable to find out, they should discuss the issue.

We thank the reviewer for reminding us about the discovery in the 60s' of four Thais and two Chilcotins with the Vel− phenotype. As suggested by this reviewer, we have included in the Discussion section the hereafter paragraph to discuss our findings as well as these historical findings from an ethnological point of view.

'Even though almost all the Vel− persons reported so far were of European descent including our cohort of 70 Vel− subjects, a few cases among other ethnicities have been reported. Four Thai persons were identified as Vel− in 1967 during an extended phenotyping of 328 blood donors from Bangkok (Chandanayingyong et al, 1967). It would be interesting to determine whether or not the Vel− phenotype in persons of Thai descent results from the same *SMIM1* disruption. Two out of 160 Chilcotin First Nations people (British Columbia, Canada) were also reported to be Vel− but this result should be interpreted with caution as the authors of this ethnologically oriented study did not mention their source of anti-Vel (Alfred et al, 1970). As a matter of fact, a similar study of 133 Penobscot Native Americans (Maine, USA) with a potent anti-Vel revealed no Vel− subjects "though two siblings were such weak positives that they were at first thought to be negative" (Allen & Corcoran, 1960).'

References
Ballif et al 2006. Is '3rd and' an error?

'Carraway KL 3rd' is indeed the abbreviated name of Dr. Kermit L. Carraway III who is co-author of 65 articles indexed in PubMed as of February 2013.

Legend to Fig. 2, one up from bottom. I suggest 'hash symbols' in place of 'number signs'.

To the best of our knowledge, 'hash symbols' and 'number signs' are synonymous terms for the typographical symbol #. We leave the decision to the copy editor to choose the most appropriate term.

Additional Author Correspondence 08 March 2013

After a delay of almost three weeks, I have finally got an accession number for the sequence of the SMIM1 gene from individuals with the Vel- blood type! This accession number (GenBank KC751412) can now be included in the Materials and Methods section of our manuscript (EMM-2013-02466-V2).

Hoping that you will make the final decision for publication soon, I thank you again for your time and consideration.
