# Supplementary material for: Disruption of SMIM1 causes the Vel− blood type
Source: EMBO Mol Med. 2013 Apr 15;5(5):751–61. doi: 10.1002/emmm.201302466 (PMC3662317; doi:10.1002/emmm.201302466)
Supplement: Supplementary file 2 [file emmm0005-0751-sd2.pdf]

Supporting Information

Disruption of *SMIM1* causes the Vel− blood type

Bryan A. Ballif, Virginie Helias, Thierry Peyrard, Cécile Menanteau, Carole Saison, Nicole Lucien, Sébastien Bourgouin, Maude Le Gall, Jean-Pierre Cartron and Lionel Arnaud

Table of Content :

- Table S1 ………….. p1

- Table S2 ………….. p2

Supporting Information

Top Hit #	PepNovo hexamer	Likely Match	Likely Protein ID
1	LTLLTV	ITLLKL	Chymomotrypsin A (*Bos taurus*)
2	LTLLTV	ITLLKL	Chymomotrypsin A (*Bos taurus*)
3	QTLAAN	QTLAAN	Chymomotrypsin A (*Bos taurus*)
4	QTLAAN	QTLAAN	Chymomotrypsin A (*Bos taurus*)
5	QTLAAN	QTLAAN	Chymomotrypsin A (*Bos taurus*)
6	QTLAAN	QTLAAN	Chymomotrypsin A (*Bos taurus*)
7	QDKTGF	QDKTGF	Chymomotrypsin A (*Bos taurus*)
8	KLSTAA	KLSTAA	Chymomotrypsin A (*Bos taurus*)
9	**GKLGLA**	**GKLGIA**	**LOC388588** (SMIM1; *Homo sapiens*)
10	QVSLQD	QVSLQD	Chymomotrypsin A (*Bos taurus*)
11	NLSLQD	QVSLQD	Chymomotrypsin A (*Bos taurus*)
12	QTLAAN	QTLAAN	Chymomotrypsin A (*Bos taurus*)
13	QTLAAN	QTLAAN	Chymomotrypsin A (*Bos taurus*)
14	**ESHVHY**	**ESHVHY**	**LOC388588** (SMIM1; *Homo sapiens*)
15	QDKTGF	QDKTGF	Chymomotrypsin A (*Bos taurus*)
16	VNWVQQ	VNWVQQ	Chymomotrypsin A (*Bos taurus*)
17	WVQQTL	WVQQTL	Chymomotrypsin A (*Bos taurus*)
18	TPATTG	TPATTG	Nup214-like (*Homo sapiens*)
19	VGLVSW	VGIVSW	Chymomotrypsin A (*Bos taurus*)
20	PVLSGL	PVLSGL	Chymomotrypsin A (*Bos taurus*)
21	VNWVGA		unknown
22	**PQESHV**	**PQESHV**	**LOC388588** (SMIM1; *Homo sapiens*)
23	GFNVLA		several
24	GSSSEK	GSSSEK	Chymomotrypsin A (*Bos taurus*)
25	VQQTLA	VQQTLA	Chymomotrypsin A (*Bos taurus*)

Table S1: Top 25 hexamers identified by PepNovo from MS/MS spectra acquired in the orbitrap of the chymotryptic peptides derived from the Vel-specific, purified 18 kDa band. See Methods for details. The hexamers are ranked by PepNovo scoring. Note that each hexamer corresponds to a single mass spectrum and that repeated sequences are the result of the same peptide ion captured in multiple MS/MS spectra or that the hexamers are common to distinct but overlapping peptide ions. Each hexamer was blasted against either the human (*Homo sapiens*) or bovine (*Bos taurus*) databases given the origins of the Vel antigen and chymotrypsin respectively. The likely match to a given protein ID was freely adjusted for the isobaric nature of leucine and isoleucine. Underlined letters are not simply explained by sequence information or mass values alone, but are the amino acids present in the Likely Protein ID.

Supporting Information

Name	Sequence	Location	Direction	Position in NC_000001.10
LOC388588-1	5' CATTCCAAAGCTCACTCACAG	5' side of exon 1	sense	3688283-3688303
LOC388588-2	5' CTGGAAGATGGTGAGGGTG	5' side of exon 1	sense	3688491-3688509
LOC388588-3	5' CTGGAGTGTGCTGGAAGGA	intron 2	sense	3691608-3691626
LOC388588-4	5' GGAATTCTCGCTTGGTCCC	intron 2	sense	3691672-3691690
LOC388588-5	5' AGAGAGGAGGCTGTAGCTG	3' side of exon 4	antisense	3692809-3692827
LOC388588-6	5' GGACACATAGCCACCCTTAC	3' side of exon 4	antisense	3693020-3693039
LOC388588-7	5' CGTCTCTCCACACTCATGT	intron 2	antisense	3689917-3689935
LOC388588-8	5' CAGCATGCAGCCCCAG	exon 3	sense	3691934-3691949
LOC388588-9	5' CGGGGCAGCATTTATTTG	exon 4	antisense	3692479-3692496
LOC388588-qPCR1	5' AGGAGAGCCACGTCCACTA	exon 3	sense	3691948-3691967
LOC388588-qPCR4	5' AGGCCTCTTCTGTGCTGGAC	exon 3	antisense	3692015-3692034

Table S2: Primers used in this study.
